# Long-Term Stable Biosensing Using Multiscale Biostructure-Preserving Metal Thin Films

**DOI:** 10.3390/bios16010063

**Published:** 2026-01-16

**Authors:** Kenshin Takemura, Taisei Motomura, Yuko Takagi

**Affiliations:** 1 Integrated Research Center for Wellbeing, National Institute of Advanced Industrial Science and Technology (AIST), Tosu 841-0052, Saga, Japan; 2 Sensing Technology Research Institute, National Institute of Advanced Industrial Science and Technology (AIST), Tosu 841-0052, Saga, Japan; t.motomura@aist.go.jp; 3 Molecular Biosystems Research Institute, National Institute of Advanced Industrial Science and Technology (AIST), Tsukuba 305-8566, Ibaraki, Japan; yuko-takagi@aist.go.jp

**Keywords:** biosensor, biostructure, infectious disease, microparticle

## Abstract

Microparticle detection technology uses materials that can specifically recognize complex biostructures, such as antibodies and aptamers, as trapping agents. The development of antibody production technology and simplification of sensing signal output methods have facilitated commercialization of disposable biosensors, making rapid diagnosis possible. Although this contributed to the early resolution of pandemics, traditional biosensors face issues with sensitivity, durability, and rapid response times. We aimed to fabricate microspaces using metallic materials to further enhance durability of mold fabrication technologies, such as molecular imprinting. Low-damage metal deposition was performed on target protozoa and Norovirus-like particles (NoV-LPs) to produce thin metallic films that adhere to the material. The procedure for fitting the object into the bio structured space formed on the thin metal film took less than a minute, and sensitivity was 10 fg/mL for NoV-LPs. Furthermore, because it was a metal film, no decrease in reactivity was observed even when the same substrate was stored at room temperature and reused repeatedly after fabrication. These findings underscore the potential of integrating stable metallic structures with bio-recognition elements to significantly enhance robustness and reliability of environmental monitoring. This contributes to public health strategies aimed at early detection and containment of infectious diseases.

## 1. Introduction

The spread of viruses and other bioparticles has caused numerous deaths, and social stagnation is inevitable in the event of a pandemic [1,2]. A major challenge is the inability to determine the distribution of fine particles that silently spread in a vast environment [3]. Microorganisms and viruses are similar in size to particulate matter, such as PM2.5, dispersed in air [4]. Advances in optical methods have made it possible to detect airborne particulates at the nanoscale; however, their effectiveness is limited because they cannot determine whether they are infectious particles [5,6].

Environmental sensing technologies have been extensively studied for the detection of viruses [7,8]. Many viruses that pose public health concerns are airborne; therefore, they can be translocated from collected aerosols into a type of solvent [9,10]. This test solution is then applicable for highly sensitive detection using a trapping substance or genetic testing techniques, such as PCR [11,12]. The physical behavior of particulates in flowing water has been well simulated and can be combined with a variety of detection systems [13,14].

Methods for detecting microorganisms and viruses can be broadly classified into genetic testing techniques and immuno-methods that use antigen–antibody reactions [15,16,17]. For genetic testing, the LAMP method is a simplified assay that enables rapid identification of viruses that have already been genetically identified [18,19]. Immuno-based detection methods have been improved to achieve PCR-level sensitivity owing to advances in nanotechnology [20,21]. Trapped substances can be loaded in nanoparticle form for chemical and electrostatic actions [22,23]. Since nanoparticles possess the ability to enhance optical and electrochemical signals, sensitivity can be improved by more than 100-fold when using nanoparticles compared to detection methods without nanoparticles [24,25]. As sensing devices, electrochemical measurement systems can be designed to be relatively small and inexpensive; therefore, highly sensitive electrochemical measurement systems are promising choices for widespread public use [26,27,28]. An ultrasensitive detection system with a sensitivity of ag/mL could be used as a virus detection system that can detect viruses in patients even in the early stages of infection by using optimal materials and detection procedures [29].

**Figure 1:**
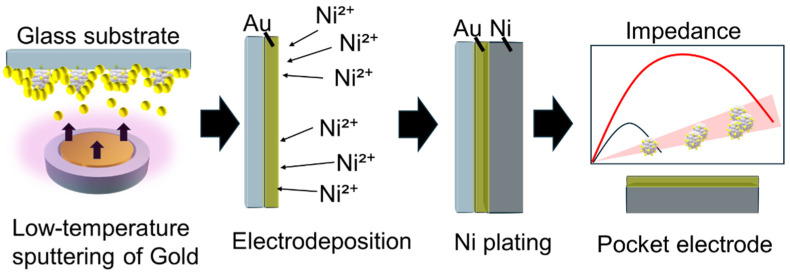
Schematic of an antibody-free biosensing electrode fabrication method using low-temperature sputtering and its measurement technique.

Traditional biosensors often rely on soft organic layers (e.g., antibodies) that can lose their activity over time, limiting their durability and reproducibility under real-world conditions. In contrast, our approach creates robust metallic microspaces that preserve the native surface morphology of bioparticles, effectively forming a physical “imprint” of the target analyte. Using low-temperature magnetron cathode deposition, we achieved rapid electron confinement and uniform film growth within 10 min following plasma ignition, enabling fast and scalable fabrication. These morphology-preserving microspaces promote selective immobilization simply by controlling droplet movement across the surface, eliminating the need for static binding or complex labeling steps. The result is metal pocketcapture method that is inherently stable, reusable, and compatible with rapid impedance readouts. By combining mechanical robustness with the selective guidance of particles into cavities, the sensor substrate supports durable and rapid biosensing suitable for pandemic-relevant surveillance (Figure 1).

## 2. Materials and Methods

### 2.1. Materials and Instruments

Phosphate-buffered saline (PBS) was procured from Wako (Osaka, Japan), and bovine serum albumin (BSA) was sourced from Sigma-Aldrich Co. LLC (Tokyo, Japan). Recombinant Norovirus GII.4VP1 virus-like particles and influenza virus A (H3N2) were purchased from Abcam (Cambridge, UK). The electroforming solution was prepared by adding 100 mL of a 50% *w*/*w* aqueous nickel (II) sulfamate solution (Thermo Fisher Scientific, Waltham, MA, USA). An electrochemical analyzer (ALS 832D, BAS Inc., Tokyo, Japan) was used to assess the electrochemical properties, and an AUTOLAB PGSTAT204 (Metrohm, Herisau, Switzerland) was used to evaluate the electrochemical sensor performance, including square-wave voltammetry and impedance measurements. Scanning electron microscopy (SEM) was conducted using a JSM-9100F microscope (JEOL Ltd., Tokyo, Japan) to examine the fabricated electrode surfaces. Surface roughness and nanoscale height were analyzed using atomic force microscopy (MFP-3D Origin+, Oxford Instruments, Buckinghamshire, UK).

### 2.2. Metal Sputtering on Bioparticles

The deposition of thin metal films on bioparticles was accomplished using a magnetic mirror-type magnetron cathode (M3C) [30,31,32]. The plasma particles were confined between two regions of strong magnetic fields, a phenomenon referred to as the magnetic mirror confinement effect. This effect inhibits the cross-field diffusion of charged particles, thereby minimizing the thermal shock to the sample surface caused by secondary electrons emitted from the gold target surface and reducing heat-induced sputtering. The sputtering process was conducted for 10 min at a pressure of 0.13 Pa (Ar, 10 sccm) with an RF input power of 30 W. The distance between the aluminum target (99.99% purity, 50 mm in diameter, and 5.0 mm in thickness) and the sample surface was maintained at 50 mm. The process gas pressure was monitored using a manometer positioned on the sidewall of the vacuum chamber. Argon gas was introduced at the top of the vacuum pump series. RF power supplies with power-control ranges of 30–100 W (T161-5313HAA, Thamway Co. Ltd., Fuji, Japan) were used. The vacuum chamber was evacuated to a pressure of less than 5 × 10^−4^ Pa.

### 2.3. Mold Plating for Electrode Fabrication

A morphology-replicating gold thin film was converted into a mechanically robust thick film by electrodepositing nickel onto the gold template. The gold film was vacuum-dried to remove residual solvent and moisture. Nickel deposition was then carried out by chronoamperometry at −1.9 V (vs. Ag/AgCl) for 3 h in a Ni^2+^ electrolyte maintained at ~75 °C. During deposition, the gold thin film served as the working electrode, a nickel plate (4N) as the counter-electrode, and Ag/AgCl as the reference electrode to ensure stable potential control. The electrochemical reduction of nickel was carried out at a constant current of 100 mA. The nickel film grew to a thickness of approximately 20 µm in three hours, imparting physical durability. The elevated bath temperature promoted rapid nucleation and dense growth, thickening the gold–nickel composite while preserving the replicated bioparticle surface morphology. After plating, the composite was detached from the glass substrate using a gentle mechanical lift-off. To remove bioparticles and residual organics from the gold surface, the film was sequentially washed with acetone and ethanol (with optional brief sonication), followed by rinsing with ultrapure water and nitrogen-drying. The resulting thickened Au–Ni electrode was then ready for subsequent impedance measurements.

### 2.4. Electrochemical Bioparticle Detection

The fabricated gold–nickel composite served as the working electrode for electrochemical impedance spectroscopy (EIS)-based measurement of bioparticles. Prior to measurement, 1 mL of the sample-containing solution was dispensed to fully cover the active area of the electrode. The droplet was gently guided across the surface to promote contact with the microspaces, and excess liquid was carefully wicked away using a Kimwipe to achieve a thin, uniform film while avoiding mechanical abrasion of the electrode. The surface was then rinsed with 1 mL of phosphate-buffered saline (PBS) to remove unbound or loosely associated material, leaving immobilized bioparticles within the cavities. EIS measurements were performed in a three-electrode configuration using a gold–nickel working electrode, a platinum counter-electrode, and a Ag/AgCl reference electrode, with 1× PBS as the supporting electrolyte. The impedance spectrum was acquired over a frequency range of 100 kHz to 0.1 Hz while applying a 0.3 V bias, and the electrical resistance was extracted from the resulting data. To improve data quality, care was taken to minimize bubbles, maintain a consistent temperature, and allow brief stabilization before each scan. Blank (PBS only) and post-rinse baseline measurements were performed to assess specificity and reusability. Between measurements, the electrode was rinsed and dried to prevent carryover, which enabled reproducible operation across multiple runs.

## 3. Results and Discussion

### 3.1. Less Sputtering Damage on Bioparticles

**Figure 2:**
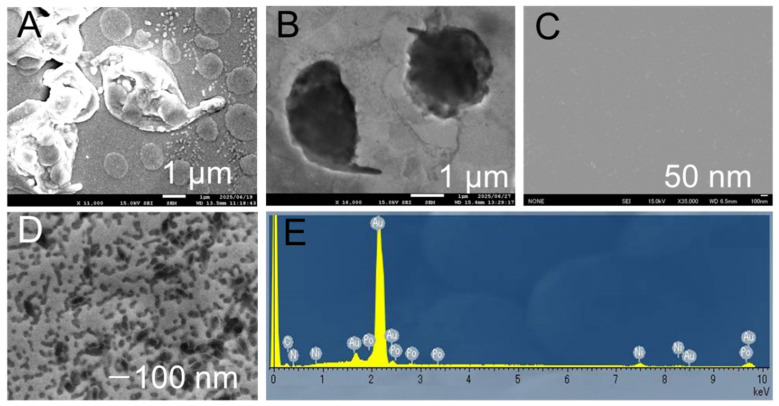
(**A**) High-magnification scanning electron microscopy image of gold-coated Ama. (**B**) High-magnification image of the gold surface morphology after thickening and removing Ama. (**C**) High-magnification image of the virus-immobilized substrate surface after gold coating. (**D**) High-magnification image of the gold surface after pocket formation and removal of Novovirus-like particles, and (**E**) its elemental analysis results. Ama, amastigote.

The fabrication of sensor electrodes using low-temperature deposition technology on bioparticles, followed by thick-film formation, was performed on particles of different size scales: protozoa and NoV-LPs. In the protozoan case, the gold pockets fabricated from the structures observed after deposition preserved even submicron-scale intact flagella. Furthermore, surface irregularities were measured based on the height differences created within the pockets (Figure 2A,B). Optical analysis of the surface height using a laser microscope before and after the deposition, and within the pockets, yielded similar numerical values. This suggests that even hydrated materials undergo minimal deformation during the deposition process, demonstrating sufficient precision to form concavities that can accommodate specific materials. Similar tests were performed on the NoV-LPs. Difficulties were encountered during post-deposition surface observation using SEM because the virus particles were buried due to the film thickness (Figure 2C). However, the examination of the delaminated surface after thickening revealed numerous depressions of approximately 30–40 nm (Figure 2D). This value aligns with typical values in the literature for NoV-LPs. These results indicate that the low-temperature deposition process can be completed without inducing structural changes in the target material owing to the rapid dehydration caused by heating or vacuum exposure, even in the presence of bioparticles. Moreover, the nickel electroforming process used for thickening suggests that even minute depressions of approximately 33 nm are unaffected by factors such as physical stress and pressure change. Elemental analysis of the NoV-LP pocket confirmed the presence of Au and Ni peaks, indicating that the electrode structure was preserved during deposition and thickening (Figure 2E). Cross-sectional SEM was performed to demonstrate the high physical durability of the samples (Figure S1). A Ni film exceeding 20 µm in thickness was formed on a gold substrate via electrodeposition. Elemental analysis suggested that the two types formed separately without alloying during the reaction. Laser microscopy analysis confirmed that the Au thin film had a thickness of approximately 350 nm, whereas the Ni film was deposited at approximately 57 times that thickness (Figure S2). The fact that such fine structures remained intact under these conditions suggests that the nickel electroforming process occurred under low-stress conditions.

### 3.2. Detection of Protozoan Using Metal Pocket

**Figure 3:**
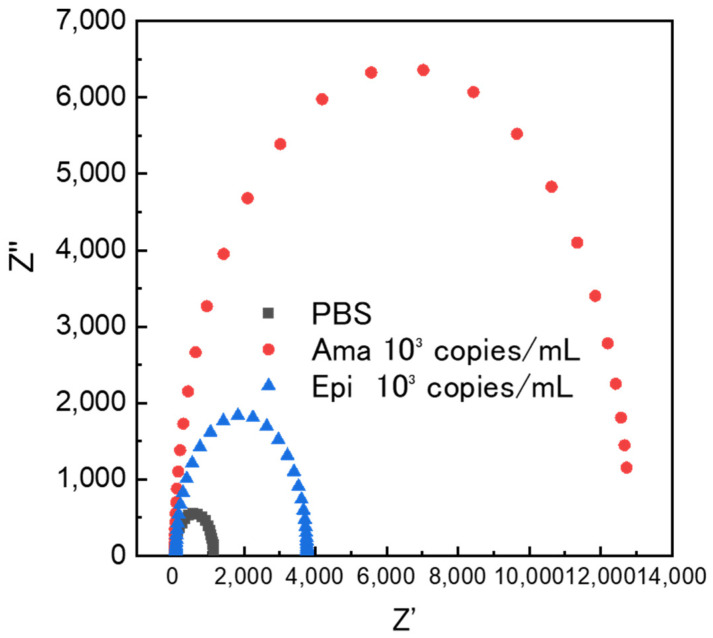
Results of measuring changes in electrical resistance using electrodes that preserve the Ama structure. Ama, amastigote; Epi, epimastigote; PBS, phosphate-buffered saline.

The protozoan parasite *Trypanosoma cruzi* undergoes morphological transitions between infection stages. The intracellular Amastigote stage (Ama) has a round cell body and has minimal flagellum. The insect stage Epimastigote (Epi) is spindle-shaped and possesses a well-developed flagellum. Electrode fabrication fromAma-stage *T. cruzi* enables differentiation of morphologically distinct stages using electrochemical signals (Figure 3). Measurements were performed using buffer solutions without protozoa and sample solutions containing 10^3^ copies/mL of the Ama andEpi stages. Post-dispersion impedance measurements revealed a significant increase in electrical resistance at the Ama stage. In comparison, the Epi stage exhibited a similar increase in the current value, albeit at a lower intensity. This was attributed to the presence of a certain number of protozoa with Ama-like morphology, even within the Epi stage culture fraction. These results suggest that µm-scale particles can be captured onto electrodes fabricated from moisture-rich microorganisms.

### 3.3. NoV-LP Detection Using Metal Pocket Electrodes

**Figure 4:**
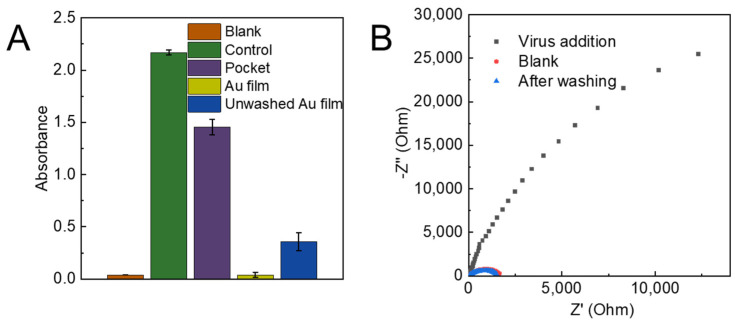
(**A**) Results of measuring changes in surface absorbance with and without NoV-LPs (*n* = 3). (**B**) Cole–Cole plot showing changes in electrical resistance measured at each step of the procedure on the substrate surface for NoV-LP measurement. NoV-LP, Novovirus-like particle.

The observed dependence of electrical resistance on droplet handling provides valuable insight into the physical mechanisms governing the entrapment of NoV-LPs within fine cavities on the electrode surface (Figure 4A). Under static conditions, where the applied 100 ng/mL NoV-LP solution was allowed to rest for 10 min, low resistance indicated negligible protein adsorption and minimal penetration of particles into the cavities. This outcome is consistent with a scenario in which the droplet exhibits limited lateral motion, weak shear at the interface, and a largely pinned contact line that does not generate sufficient convective transport to enter the nano-size hole for NoV-LPs. Given that NoV-LPs typically carry a net negative surface charge at neutral pH, and many oxide or polymeric sensor substrates are also negatively charged in aqueous media, the electrical double layer can contribute to near-surface repulsive interactions that, in the absence of motion, favor a sparse distribution of particles on the topography rather than deep entrapment.

In contrast, the blotting procedure using Kimwipes introduces controlled droplet displacement across the sensor, thereby imposing lateral shear, transient pressure gradients at the moving meniscus, and sustained convective transport. These hydrodynamic conditions can actively guide particles toward and into the micro/nanocavities (Figure S3). The moving contact line acts as a conveyor of particles. Near the three-phase boundary (liquid/air/solid), capillary forces and local flow acceleration increase the probability of particles entering apertures whose size is commensurate with or slightly larger than the particle diameter. As the droplet traverses the surface asperities, pressure-driven infiltration can occur; once particles cross the cavity mouth, capillary confinement and steric hindrance synergize to retain them. The marked increase in electrical resistance after the blotting and rinsing steps suggests that the physical presence of NoV-LPs within the cavities partially obstructs the ionic pathways and increases the tortuosity, thereby reducing the effective ionic conduction. The increase in resistance is consistent with a microstructural transition from open, conductive pores to partially occluded pores, where the current lines are forced to detour around the embedded particles.

The further amplification of resistance under fine vibrations underscores the role of dynamic fluid motion—beyond simple wiping—in promoting entrapment. Vibrational excitation can induce microstreaming within the droplet, repeatedly depinning and repinning the contact line, and enhancing the recirculating flows. These oscillatory motions increase the frequency of particle–cavity encounters, and the repeated meniscus sweeps across the microfeatures facilitate more efficient guidance of VLPs into the recesses. In addition, vibration can reduce the boundary layer thickness near the surface, thereby diminishing hydrodynamic screening and improving delivery to the cavity openings. These results support an operational protocol in which the droplet is deliberately translated over the sensor surface (either by gentle blotting or controlled vibration) before removal, rather than allowing it to rest statically. The entire procedure is completed in approximately 1 min, providing a rapid, low-power approach that is favorable for point-of-care or field measurements.

Importantly, the demonstration of reusability, where cleaning with ultrapure water returns the resistance to a value approximating the blank, supports the interpretation that the dominant mechanism is physical embedding rather than irreversible adsorption or chemical binding (Figure 4B). If adsorption is prevalent, one would expect persistent resistance changes even after rinsing, as a monolayer or multilayer of protein could remain on the cavity walls. The recovery toward the baseline suggests that the VLPs can be dislodged and flushed from the cavities under appropriate rinsing conditions, enabling multiple measurement cycles with the same substrate. This reusability can be strengthened by optimizing the cleaning protocol, for example, by evaluating the effect of rinse volume, flow rate, and gentle orthogonal agitation, limiting surfactants to very low concentrations if needed to avoid altering surface chemistry, and verifying that the substrate’s native wettability and charge properties are restored after each cycle. AFM analysis also confirmed that NoV-LPs remained on the metal pocket even after washing (Figure S4). The AFM results showed that prior to NoV-LP addition, there were holes of approximately 30 nm on the surface, while after addition, an amorphous protein-like substance was observed on the surface. These findings indicate that the NoV-LPs immobilized in the pocket were not removed during washing.

Although these results are compelling, several considerations can further refine our mechanistic understanding of them and improve their robustness. First, controls are essential to rule out artifacts introduced by the blotting material; cellulose fibers or trace additives from tissues could, in principle, be deposited on the surface and elevate resistance. Blank tests, in which the same blotting and vibration steps are performed with pure water and alternative inert wipers or spreaders, should be compared to ensure that the signal change is truly due to VLP entrapment. Second, complementary imaging (SEM, AFM, and confocal fluorescence with labeled VLPs) would provide direct evidence of particle localization within cavities and help correlate occupancy with electrical changes. Third, impedance spectroscopy across frequency (rather than a single resistance readout) would allow the deconvolution of changes in pore conduction versus interfacial polarization; for instance, increased low-frequency resistance coupled with altered capacitance would support the physical occlusion of conductive pathways alongside modifications to the double layer.

The sensor performance can be tuned by adjusting the solution conditions and operational parameters. Ionic strength modulates the screening of electrostatic interactions; moderate salt concentrations may reduce repulsion and enhance penetration, whereas excessively high ionic strengths can promote nonspecific aggregation and clogging. The pH affects both VLP and surface charge, potentially shifting the balance between repulsion and attraction. Droplet volume, travel speed during blotting, wiping directionality relative to cavity orientation, and vibration frequency/amplitude are practical variables that likely exhibit optimal ranges for efficient trapping without overpacking. Environmental factors such as humidity and temperature influence the evaporation rates and Marangoni flows that accompany concentration gradients within the droplet; maintaining consistent ambient conditions will improve reproducibility. Geometry is also important: cavity diameter, depth, entrance shape, and pitch govern hydrodynamic access and retention forces; systematic variation of these features could identify designs that maximize capture efficiency while preserving sensor reusability.

Finally, translating this entrapment mechanism into virus detection workflows suggests several strategies for future research. If the VLPs used here are surrogates for intact virions, calibration across clinically relevant concentrations will establish the dynamic range and limit of detection (LOD). If the sensor relies on entrapment followed by specific recognition (e.g., antibodies or aptamers lining the cavities), optimizing the surface chemistry to balance physical guidance with biochemical selectivity becomes critical. Matrix effects from real samples (stool, saliva, environmental water) can be assessed by spiking controls to quantify non-specific fouling and define appropriate pre-treatment or blocking steps. Overall, the rapid increase in resistance achieved by controlled droplet motion and vibration, combined with demonstrable reusability after rinsing, positions this approach as a practical, scalable, and instrument-free method for high-throughput screening and field-deployable measurements.

**Figure 5:**
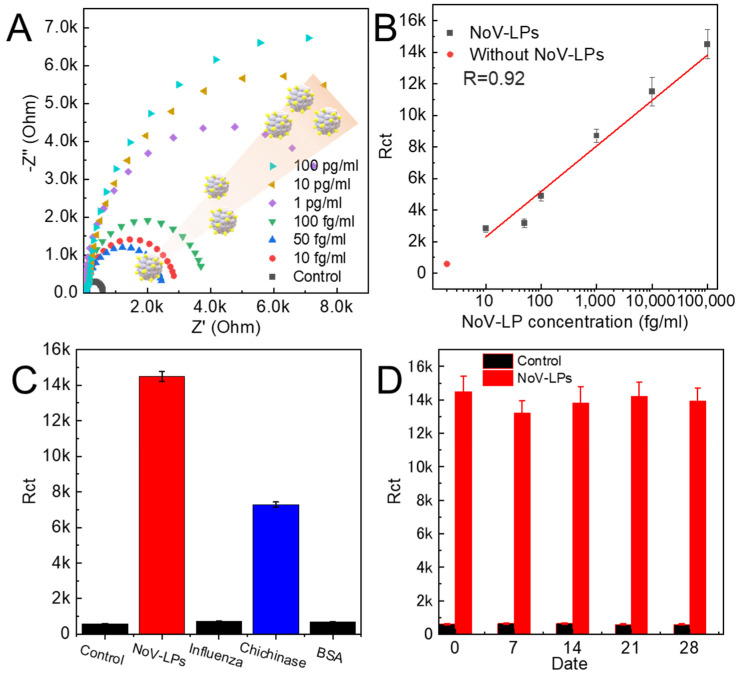
(**A**) Results of measuring concentration-dependent changes in electrical resistance for NoV-LPs. (**B**) Calibration curve created from electrical resistance values (*n* = 3). (**C**) Selectivity verification test. Results measured at a concentration of 100 ng/mL for all compounds except NoV-LPs (*n* = 3). (**D**) Sensor stability evaluation test results. BSA, bovine serum albumin; NoV-LP, Novovirus-like particle (*n* = 3).

A NoV-LP detection test was performed to evaluate the quantitative capability of the sensor. A significant increase in electrical resistance was observed from a low concentration of NoV-LP solution (10 fg/mL to 100 pg/mL) (Figure 5A,B). Furthermore, the calibration curve constructed from the repeated tests showed a linear relationship, indicating that the sensor provided reliable quantitative performance across a wide dynamic range. Compared to conventional immunosensors, such as ELISA, the most significant advantage is the elimination of the waiting time for antibody binding reactions, enabling highly sensitive measurements by simply removing the solution. This rapid detection capability is particularly advantageous for point-of-care applications and environments in which time-sensitive diagnostics are critical. The detection limit obtained from the calibration curve was 0.84 fg/mL, demonstrating an exceptionally high sensitivity suitable for early-stage viral detection. Furthermore, when evaluating the selectivity of this sensor substrate using a 100 pg/mL NoV-LP solution, the reaction of the sensor substrate with other solutions containing the influenza virus (IFV) at 100 ng/mL using the same procedure resulted in an increase in electrical resistance only for chitinase (Figure 5C). This may be because, compared to the other samples, IFV has an estimated size of 80–120 nm, which does not match the pocket size, whereas chitinase has an estimated size of 10–12 nm based on its molecular weight, which is similar to that of NoV-LPs [33,34]. Considering that proteins around 5–10 nm, such as BSA, which has a non-particle shape, may exhibit similar reactions, a more intensive process tailored to the characteristics of impurities in the target sample is required to enhance the substrate selectivity. This can be achieved through additional washing steps with ultrapure water after sample loading or by introducing surface modifications that reduce non-specific adsorption.

To further assess the robustness of the sensor, stability tests were performed by storing the sensors in an oven maintained at 70 °C and conducting detection tests for 100 pg/mL of NoV-LPs every 7 days (Figure 5D). The results showed a maximum decrease in the sensor performance of approximately 10% as the resistance value increased slightly; however, this was suggested to be within the margin of error relative to the standard deviation. These findings indicate that the sensor maintains its functional integrity under accelerated aging conditions, suggesting long-term usability in practical applications. The advantage of this sensor lies in its unique design, which does not require capture agents such as antibodies to achieve selectivity. Instead, its performance depends on the intrinsic structural properties and strength of the metal. Therefore, the sensor can be considered fundamentally reusable, provided that the surface structure remains intact and is not damaged. This characteristic significantly reduces operational costs and simplifies maintenance compared to conventional biosensors, making it an attractive solution for scalable deployment in clinical and environmental monitoring.

We compared the performance of this detection system with examples from the latest research (Table 1). Regarding sensitivity, it is evident that this approach achieves sufficiently high sensitivity even when compared with recent studies. Metal pockets can be detected without compromising the high sensitivity of electrochemical sensors owing to their high conductivity. Furthermore, the time required to complete the measurements demonstrated performance comparable to that of the sensor groups using aptamers. Its rapidity is also comparable to that found in recent reports, and considering the stability of the sensor itself, it demonstrates potential as an effective biosensor.

## 4. Conclusions

Integrating stable metallic architectures with biorecognition-inspired transduction yields a durable, reagent-free sensing platform that maintains its performance under accelerated aging and eliminates cold-chain constraints. Simple fabrication, low operating costs, and reusability via metal recollection enable scalable, continuous, and on-site monitoring in harsh or resource-limited settings. These attributes position this technology as a promising solution for proactive public health surveillance, early warnings, and rapid interventions across healthcare facilities, food and water systems, and urban environments, while also extending to clinical triage and environmental risk assessments. This approach is compatible with mass manufacturing and supports rapid measurements without specialized infrastructure, establishing a practical foundation for population-scale networked biosensing.

The key merits of the proposed method include durability and stability with minimal performance drift under storage and field conditions, reagent-free operation that simplifies workflows and reduces maintenance, sustainability through reusable metal components, scalability via micro/nanofabrication and roll-to-roll processes, field-readiness for rapid deployment, suitability for continuous surveillance and early warning networks, and broad applicability spanning public health, food and water safety, clinical decision support, and environmental risk monitoring.

Future studies should focus on enhancing selectivity and antifouling by optimizing surface nano/microstructures and coatings to suppress non-specific adsorption and sharpen discrimination among target particles and contaminants; establishing quantitative calibration protocols, reference materials, cross-reactivity libraries, and models of particle–surface interactions to refine limits of detection, specificity, and reproducibility; integrating IoT connectivity and edge/AI analytics for real-time aggregation, privacy-preserving inference, interoperable data formats, and predictive epidemiology; validating performance through multisite field trials to assess reliability, maintenance cycles, and lifecycle sustainability; advancing compact form factors, rugged packaging, and low-power or energy-harvesting modules for dense deployments; and aligning cybersecurity, data governance, and regulatory pathways to enable trusted, large-scale adoption of this technology.

## Figures and Tables

**Table 1:** Comparison of virus sensors.

Platform/Method	Target Virus/Analyte	LoD (g/mL)	Time-to-Result	Ref
NoV-LP impedance sensor (this work)	Norovirus-like particles (NoV-LPs)	0.8 fg/mL	≤10 min	—
Fiber-optic evanescent-wave (FOEW) aptasensor	SARS-CoV-2 (inactivated virus)	740 fg/mL	6 min	[35]
Electrochemical impedance (EIS) aptasensor	SARS-CoV-2 (inactivated virus)	5.1 fg/mL	NR	[36]
Paper-based fluorescent aptasensor	SARS-CoV-2 S1 protein	0.067 ng/mL (67 pg/mL)	~25 min	[37]
Fluorescent LFIA + low-cost reader	Influenza A antigen	2.91 ng/mL	≤15 min	[38]
Silicon microring resonator (Si-MRR) biosensor	SARS-CoV-2 nucleocapsid protein	~10 pg/mL (*potential sensitivity*, *preliminary*)	<10 min	[39]
PARIHN fluorescent label immunochromatography	SARS-CoV-2 nucleoprotein	9.45 pg/mL (ICAs)	NR	[40]

## Data Availability

Data supporting the findings of this study are available from the corresponding author upon reasonable request.

## Supplementary Materials

The following supporting information can be downloaded at: https://www.mdpi.com/article/10.3390/bios16010063/s1, Figure S1: Cross-section SEM observation and elemental analysis results of Au-Ni; Figure S2: (a) Observation image of the Au thin film after deposition using a laser microscope. (b) Height analysis results for film removal area and deposition area, and (c) graph of obtained film thickness; Figure S3: Impedance signal change depends on controlling of samples using different solution control methods (*n* = 3); Figure S4: Analysis results of metal pocket surface condition before and after NoV-LP addition using AFM.

## Abbreviations

The following abbreviations are used in this manuscript:

Ama	Amastigote
BSA	Bovine serum albumin
Epi	Epimastigote
NoV-LPs	Novovirus-like particles
PBS	Phosphate-buffered saline
SEM	Scanning electron microscopy
