# Supplementary material for: Long-Term Stable Biosensing Using Multiscale Biostructure-Preserving Metal Thin Films
Source: Biosensors (Basel). 2026 Jan 16;16(1):63. doi: 10.3390/bios16010063 (PMC12838530; doi:10.3390/bios16010063)
Supplement: Supplementary file 1 [file biosensors-16-00063-s001.zip › biosensors-4079290-supplementary.pdf]

Article

Long-Term Stable Biosensing Using Multiscale Biostructure-Preserving Metal Thin Films

Kenshin Takemura [1,2,*], Taisei Motomura [2] and Yuko Takagi [3]

1 Integrated Research Center for Wellbeing, National Institute of Advanced Industrial Science and Technology (AIST), Tosu 841-0052, Saga, Japan
2 Sensing Technology Research Institute, National Institute of Advanced Industrial Science and Technology (AIST), Tosu 841-0052, Saga, Japan; t.motomura@aist.go.jp
3 Molecular Biosystems Research Institute, National Institute of Advanced Industrial Science and Technology (AIST), Tsukuba 305-8566, Ibaraki, Japan; yuko-takagi@aist.go.jp
* Correspondence: takemura.kenshin@aist.go.jp

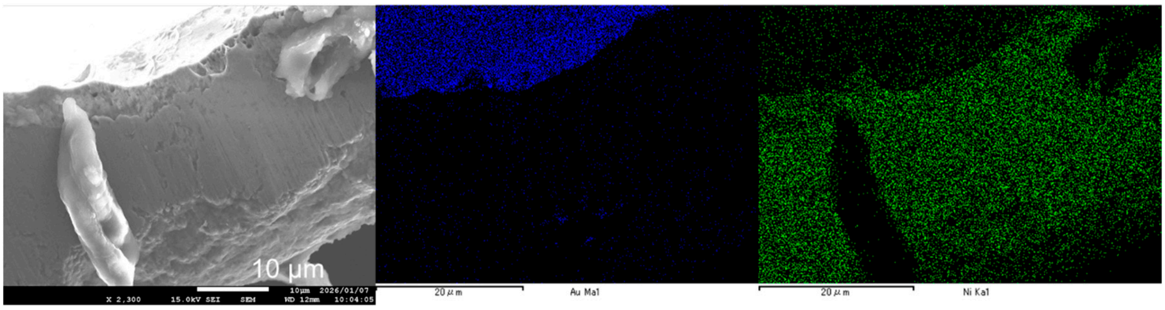

Figure S1. Cross-section SEM observation and elemental analysis results of Au-Ni.

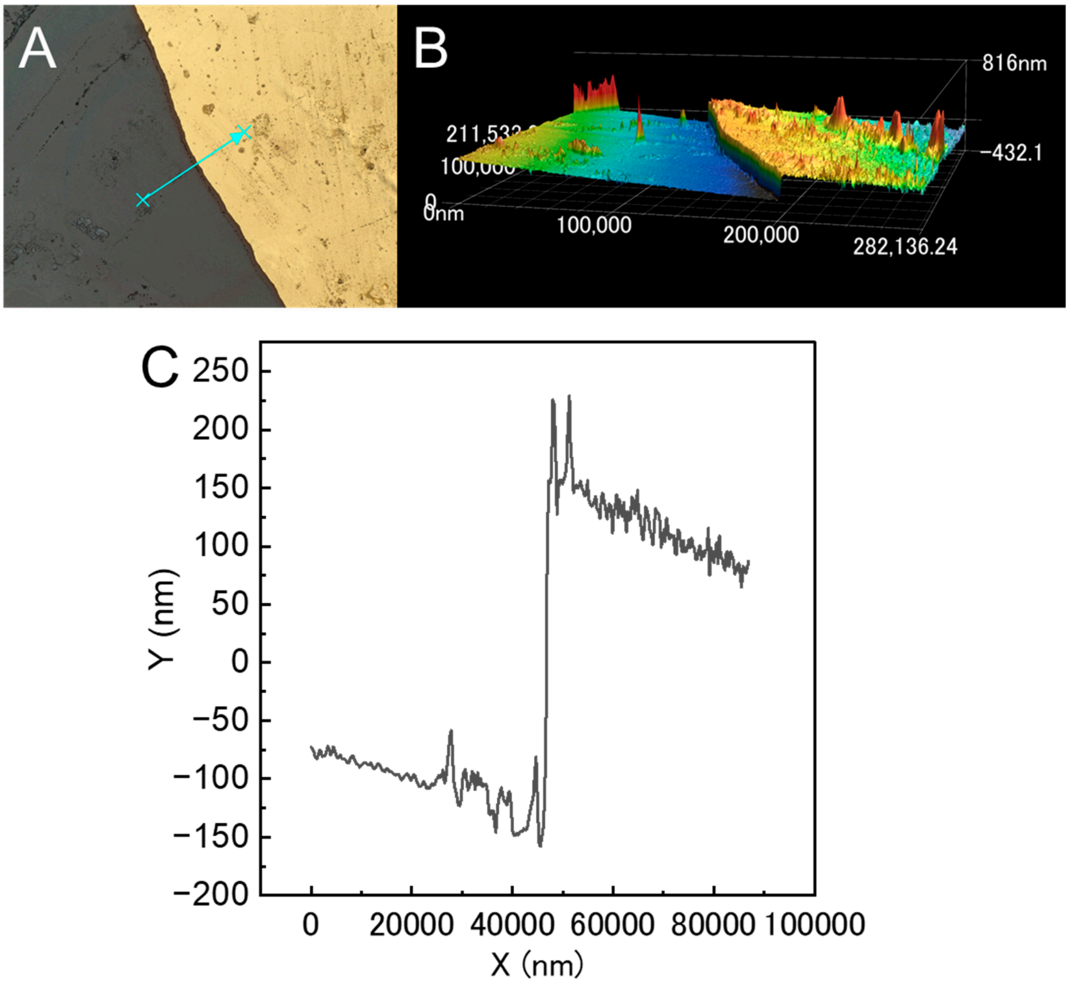

Figure S2. (a) Observation image of the Au thin film after deposition using a laser microscope. (b) Height analysis results for film removal area and deposition area, and (c) graph of obtained film thickness.

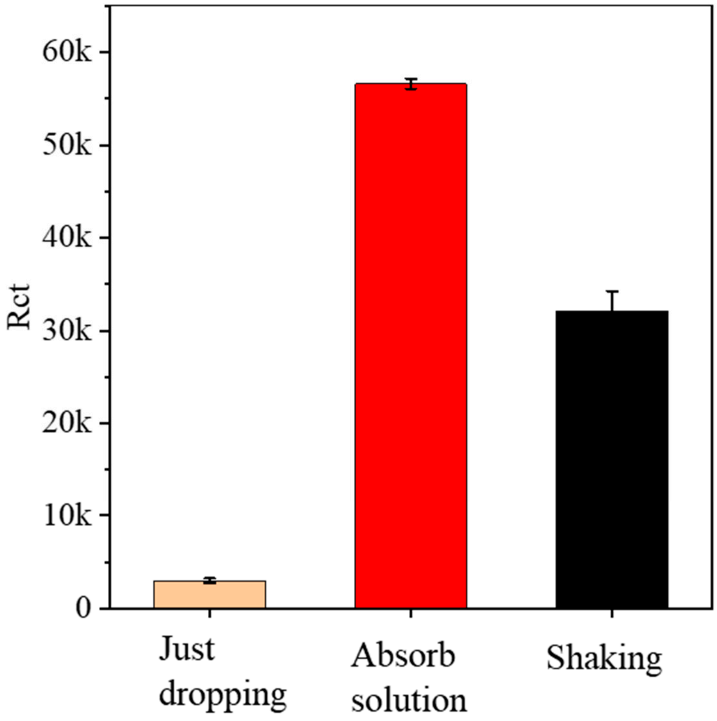

Figure S3. Impedance signal change depends on controlling of samples using different solution control methods (n = 3).

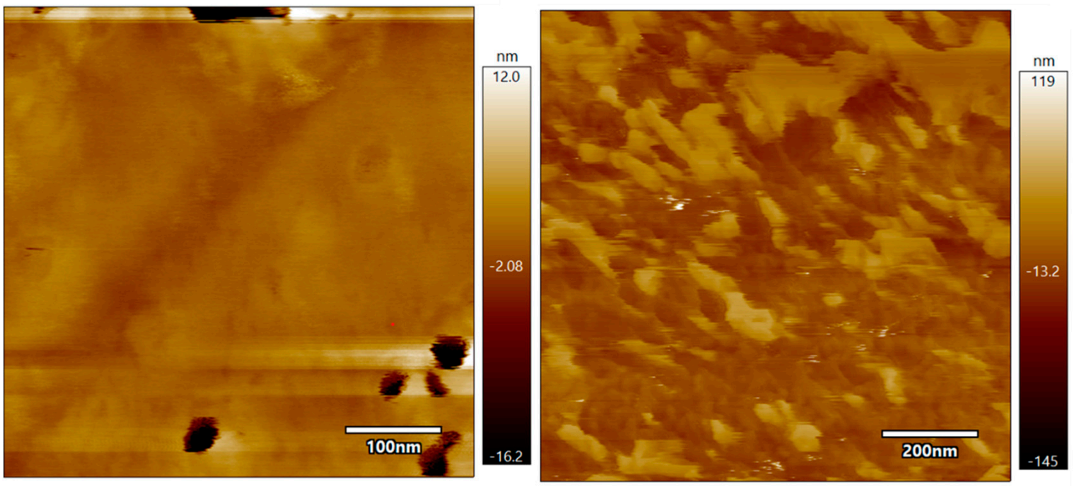

Figure S4. Analysis results of metal pocket surface condition before and after NoV-LP addition using AFM.
